# Supplementary material for: Spatio-Temporal Gait Parameters of Hospitalized Older Patients: Comparison of Fallers and Non-Fallers
Source: Int J Environ Res Public Health. 2023 Mar 4;20(5):4563. doi: 10.3390/ijerph20054563 (PMC10001499; doi:10.3390/ijerph20054563)
Supplement: Supplementary file 1 [file ijerph-20-04563-s001.zip › ijerph-2218917-supplementary.pdf]

Supplementary Material Table S1: Normative Data (Mean±SD) for Parameters Constituting Rhythm, Phases, Variability, Pace and Base of Support Domains of Gait Performance by Gender and Age **[13]**

Parameter	Men (n=108)				Women (n=186)			
	70-74	75-79	80-84	85+	70-74	75-79	80-84	85+
	n=27	n=30	n=37	n=14	n=33	n=77	n=43	n= 33
**Cadence (steps/min)**	102±8	106±10	103±8	102±11	113±20	114±13	110±9	108±10
**Step Time (s)**	0.59±.05	0.56±.05	0.59±.04	0.59±.08	0.53±.06	0.53±.06	0.55±.05	0.56±.05
**Swing (% cycle)**	36.6± 1.5	36.7±1.5	36.6±2.8	35.1±2.69	36.6±2.6	36.1±3.0	35.5±2.5	35.7±2.6
**Stance (% cycle)**	63.2±2.1	64.0±2.5	63.8±2.7	64.9±2.7	63.3±3.1	63.9±3.0	64.5±2.6	64.5±2.5
**Single Support (% cycle)**	37.1±1.8	37.0±1.7	36.5±2.2	35.2±2.1	37.0±3.20	35.8±4.8	35.6±2.4	35.7±2.8
**Double Support (% cycle)**	26.3±3.0	26.5±2.3	27.4±4.7	30.3±3.5	27.14±4.0	28.4±6.4	29.0±4.6	28.7±4.8
**Gait Speed (cm/s)**	117±16	122±15	112±17	101±22	116±20	112±17	101±15	98±20
**Step Length (cm)**	69±8	68±7	65±8	59±10	61±9	59±7	55±7	54±9
**Stride Length (cm)**	139±14	137±12	131±17	119±21	123±17	118±15	111±14	109±18
**Base of Support (cm)**	9.7±3.0	8.9±5.2	11.2±4.0	9.9±4.8	7.0±3.5	7.7±4.0	7.9±4.1	9.1±2.6
**Variability**								
**Step Length (%CV)**	4.6±6.7	5.4±2.7	5.1±2.8	5.8±3.4	7.7±11.6	5.7±7.2	5.9±2.7	6.2±2.4
**Step Time (%CV)**	5.2±6.9	4.1±2.9	4.7±2.0	5.0±1.7	7.1±8.6	5.9±6.4	5.5±2.6	5.5±2.6
**Stride Length (%CV)**	2.9±1.1	4.2±4.6	3.8±2.0	5.7±3.2	4.1±4.7	4.7±5.6	4.3±2.1	5.2±5.2
**Swing Time (%CV)**	4.5±2.2	4.5±7.8	5.1±2.2	8.6±11.4	6.2±10.5	8.5±9.5	6.2±2.2	8.0±9.7
**Stance Time (%CV)**	4.9±4.6	5.9±7.0	4.7±3.0	5.3±2.9	5.7±4.4	5.3±5.1	5.3±2.9	5.6±4.1
**Stride Speed (%CV)**	5.0±2.9	5.5±4.4	5.5±3.2	7.3±3.8	5.6±3.4	5.5±2.7	6.8±3.2	6.9±3.3
